# Impact of Breast Cancer on Ovarian Function: Dysregulation of Cholesterol Homeostasis in Cumulus Cells and Follicular Fluid

**DOI:** 10.3390/cancers18091451

**Published:** 2026-05-01

**Authors:** Alice Poitrinal, Léa Dupont, Sandra Dollet, Anja Kerksiek, Laure Chaput, Bruno Pereira, Cécily Lucas, Ludivine Riche, Lucie Chansel-Debordeaux, Marie Prades, Dieter Lütjohann, Gaëlle Marteil, Florence Brugnon

**Affiliations:** 1 Service d’Assistance Médicale à la Procréation-CECOS, CHU Estaing, 63100 Clermont-Ferrand, France; alice.poitrinal@gmail.com (A.P.); lchaput1@chu-clermontferrand.fr (L.C.); crodrigues@chu-clermontferrand.fr (C.L.); lriche1@chu-clermontferrand.fr (L.R.); fbrugnon@chu-clermontferrand.fr (F.B.); 2 Imagerie Moléculaire et Stratégies Théranostiques, Inserm, UMR 1240, Université Clermont Auvergne, 63001 Clermont-Ferrand, France; lea.dupont@doctorant.uca.fr (L.D.); sandra.carlet_dollet@uca.fr (S.D.); 3 Institute of Clinical Chemistry and Clinical Pharmacology, University Hospital Bonn, 53127 Bonn, Germany; anja.kerksiek@ukbonn.de (A.K.); dieter.luetjohann@ukbonn.de (D.L.); 4 Biostatistics Unit (DRCI), CHU Gabriel Montpied, 63000 Clermont-Ferrand, France; bpereira@chu-clermontferrand.fr; 5 Service d’Assistance Médicale à la Procréation-CECOS, Centre Aliénor d’Aquitaine, CHU de Bordeaux, 33000 Bordeaux, France; lucie.chansel-debordeaux@chu-bordeaux.fr; 6 U1312-BRIC Team Biotherapies Genetics and Oncology-BioGO, Bordeaux University, 33000 Bordeaux, France; 7 Service d’Assistance Médicale à la Procréation-CECOS, Hôpital Tenon, Assistance Publique-Hôpitaux de Paris, Sorbonne Université, 75020 Paris, France; marie.prades@aphp.fr

**Keywords:** oncofertility, breast cancer, ovarian stimulation, cholesterol biosynthesis, fertility preservation

## Abstract

**Background/Objectives**: Women undergoing fertility preservation by oocyte vitrification before oncological treatments often have a poorer ovarian response to stimulation than healthy women. This indicates that cancer itself can adversely affect ovarian function. However, this impact remains unclear, and its underlying mechanisms are poorly understood. We investigated in this study whether breast cancer, the most common form of cancer in women of reproductive age, alters ovarian function by itself. **Methods**: For this purpose, we compared the ovarian response to hormonal stimulation in women with breast cancer undergoing oocyte cryopreservation with that in oocyte donors, adjusting for age and BMI. We analysed our data according to the molecular subtype of breast cancer, tumour grade, lymph node invasion, and BRCA1 mutation status. Secondly, we evaluated whether breast cancer alters cholesterol homeostasis in cumulus cells, given its essential role in oocyte quality. The expression of genes involved in cholesterol biosynthesis was analysed in cumulus cells using RT-qPCR, while the concentrations of cholesterol and its intermediates were quantified in follicular fluid using GC-FID and GC-MS/SIM. **Results**: Compared with oocyte donors, breast cancer patients exhibited a significant decrease in collected oocytes after stimulation. At the molecular level, our data revealed a significant deregulation of cholesterol biosynthesis gene expression in cumulus cells of women with breast cancer. The quantification of cholesterol and its intermediates in follicular fluid revealed altered concentrations in women with breast cancer, suggesting a disrupted follicular microenvironment. **Conclusions**: These findings suggest that breast cancer impairs ovarian function, at least in part, by disrupting cholesterol homeostasis, which can lead to reduced oocyte competence.

## Simple Summary

Breast cancer is the most common cancer in women of reproductive age. As cancer treatment can affect reproductive function, many women opt to preserve their fertility by freezing their gametes (i.e., mature oocytes) after ovarian hormonal stimulation. However, women with breast cancer seem to produce fewer oocytes than cancer-free women, even before treatment begins. This suggests that the cancer itself may already affect fertility, although the reasons for this are unclear. This study examined whether breast cancer affects women’s response to ovarian stimulation. We also investigated the potential molecular mechanisms underlying this alteration, focusing on cholesterol, an essential lipid for oocyte quality. For this, we analysed the cells and fluid that surround the oocyte inside the ovary. Our findings highlight that breast cancer appears to be linked to changes in cholesterol regulation around the oocyte, which may partially explain the reduced ovarian response to stimulation observed in breast cancer patients.

## 1. Introduction

Recent advances in cancer diagnosis and treatments have improved the survival rate for patients [1]. However, some cancer treatments are potentially gonadotoxic and may compromise the chances of conceiving in the future [2]. As post-remission quality of life is a key concern, fertility preservation (FP) options should be offered to patients before initiating any gonadotoxic anticancer therapy [3]. For adult women, one of the standard techniques for FP is cryopreservation of mature oocytes, which is performed after controlled ovarian stimulation [4].

In the context of oncofertility, several studies have shown that the ovarian response to hormonal stimulation may be altered in cancer patients compared to cancer-free women, even before anticancer treatments are initiated [5]. Indeed, although higher doses of gonadotropins are usually administered to cancer patients [6,7,8,9], fewer oocytes are retrieved [5,10]. Furthermore, the oocyte quality of these women appears to be compromised, as they have higher rates of immature, atretic and morphologically abnormal oocytes than cancer-free women [6,7,11]. Overall, these studies suggest that the cancer itself exerts a deleterious impact on ovarian function. This was particularly suggested for breast cancer (BC), which is the most common cancer in women of childbearing age, with triple-negative BC (TNBC) patients showing the worst ovarian response to stimulation [12]. Several tumour characteristics may influence the ovarian response to stimulation, such as tumour grade and stage, or, in the case of BC, the presence of a breast cancer 1 (BRCA1) mutation [8,13,14]. However, studies on fertility in cancer patients before the onset of cancer therapy are often contradictory and really scarce [15,16]. This may be because neoadjuvant ovarian stimulation in BC patients, performed while the tumour is still present, has only recently become more common in clinical practice. This creates new opportunities to improve our understanding of oncofertility [17,18].

The mechanisms underlying the putative deleterious impact of cancer on ovarian function, and, more specifically, on the biological processes regulating folliculogenesis and oogenesis, remain unknown. One crucial process for ovarian cell function is maintaining cholesterol homeostasis, because cholesterol is the precursor to all steroid hormones, including oestrogens. Interestingly, follicular fluid meiosis-activating sterol (FF-MAS), an intermediate in the cholesterol biosynthetic pathway that is active in granulosa cells, has been shown to promote oocyte maturation in both mice and humans [19,20,21,22,23,24]. In addition, an excess of cholesterol has been shown to trigger premature oocyte activation in mice [25,26,27,28]. However, despite the well-established link between cholesterol metabolism and cancer [29], no study has ever examined cholesterol homeostasis in the ovaries of BC patients.

The aim of this study is to evaluate the possible impact of BC on ovarian function. For this purpose, we compared ovarian reserve parameters and ovarian response to hormonal stimulation in women with BC and oocyte donors (control population). These parameters were analysed according to the BC molecular subtype, tumour grade, lymph node involvement, and BRCA1 mutation status. We then investigated whether the deleterious impact of BC on the ovarian response to hormonal stimulation could be, at least partially, linked to an alteration in cholesterol homeostasis in granulosa cells. To achieve this, we compared the expression levels of eleven genes that code for enzymes and regulators of cholesterol biosynthesis in the cumulus cells (CC) of BC patients and oocyte donors by RT-qPCR: i) six important enzymes of cholesterol biosynthesis, including the two rate-limiting enzymes (HMG-CoR and SQLE) and enzymes that catalyse the formation of FF-MAS and cholesterol (CYP51, DHCR7, DHCR24); and ii) the main regulators of cholesterol biosynthesis (SREBP2, SCAP, INSIG, LXRα and LXRβ). Second, we quantified cholesterol and its intermediates in follicular fluids (FF) in BC women and oocyte donors by GC-FID and GC-MS/SIM. For this, we selected intermediates from the two pathways leading to the formation of cholesterol: the Bloch pathway (lanosterol, FF-MAS, and desmosterol) and the Kandutsch–Russell pathway (dihydrolanosterol, dihydro-FF-MAS, dihydro-T-MAS, and lathosterol).

## 2. Materials and Methods

### 2.1. Study Design and Population

**Figure 1:**
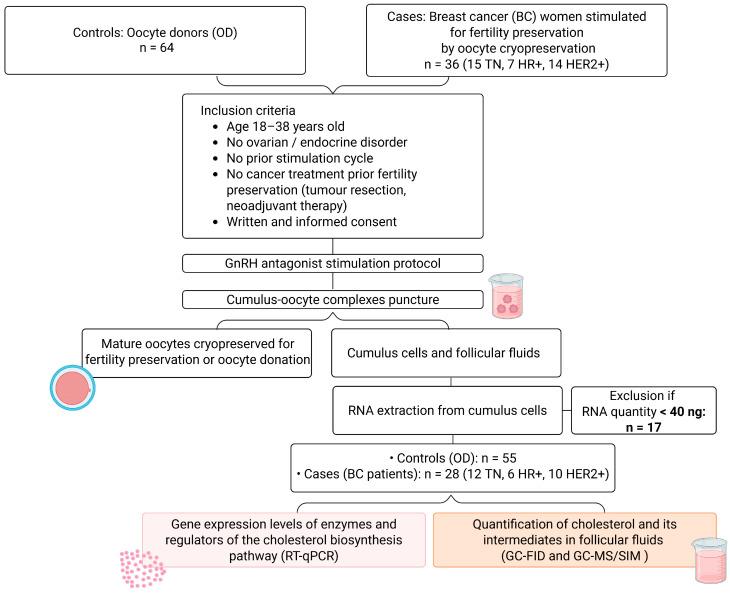
Experimental design. Thirty-six patients with breast cancer (BC) and sixty-four oocyte donors (OD) undergoing controlled ovarian stimulation for fertility preservation and oocyte donation, respectively, were included in our study. After cumulus–oocyte complex retrieval, cumulus cells and follicular fluids were isolated from each patient. RNA was extracted from cumulus cells, and patients were considered eligible for further analysis if the RNA quantity exceeded 40 ng. Subsequent analyses (highlighted in pink and orange rectangles) were performed in BC women and OD samples with sufficient RNA from cumulus cells. TN: triple-negative; HR+: hormone receptor–positive; HER2+: HER2 amplified.

This prospective, multicentric, and non-interventional study was conducted between January 2019 and May 2025 at the Assisted Reproductive Technology (ART) and FP units of Clermont-Ferrand, Bordeaux, and Paris Tenon University Hospitals, France. This study included thirty-six women (aged 18–38, Figure 1; see Table 1 for patient characteristics) with BC (BC group), who were referred to FP units for oocyte cryopreservation. Patients who had received cancer treatment (tumour resection or neoadjuvant chemotherapy) prior to FP, or who presented with any ovulatory disorder (e.g., polycystic ovary syndrome or premature ovarian failure), or who developed ovarian hyperstimulation syndrome (OHSS) following ovarian stimulation, were excluded from this study. As a control population, sixty-four oocyte donors (ODs) (aged 18–38 years; see Table 1 for OD characteristics) were included in this study. For all patients/donors, only the outcomes of the first ovarian stimulation cycle were taken into account (see Figure 1). Only BC patients and ODs for whom a sufficient RNA quantity could be extracted from the cumulus cells were included in the molecular analyses (exclusion criterion: <40 ng), leading to 55 women being included in the OD group and 28 in the BC group (see Figure 1).

### 2.2. Breast Cancer Patient Classification

BC patients were classified according to the molecular status of their tumour, as determined by immunohistochemistry: the presence or absence of oestrogen receptor (ER), progesterone receptor (PR) and amplification of human epidermal growth factor receptor 2 (HER2+). Of the thirty-six patients with BC included in this study, fifteen had TNBC (ER−/PR−/HER2−), seven had hormone receptor-positive (HR+) BC (ER+ and/or PR+) and fourteen had HER2-positive BC (HER2+, all ER+ and/or PR+). Tumours were staged using the Tumour, Node and Metastasis (TNM) classification [30] and graded according to the modified Scarff-Bloom-Richardson histologic score [31]. For the grade-based comparison, BC patients were divided into two subgroups: Grade 2 and Grade 3, since none of the BC patients presented with a Grade 1 tumour. For the comparison according to tumour dissemination, we opted for a classification based on lymph node status rather than stage, as this provides a consistent and clinically relevant measure of cancer spread, given the heterogeneous distribution of tumour stages in our cohort (see Table 2). Patients were classified into two subgroups: localised (no lymph node involvement, N0) and disseminated (with lymph node involvement, N1–N3) [30]. A search for pathogenic variants of the BRCA 1/2 gene was carried out on blood samples.

### 2.3. Oocyte, Cumulus Cells and Follicular Fluid Collection

Prior to ovarian stimulation, antral follicular count (AFC) and serum anti-Mullerian hormone (AMH) levels were determined using transvaginal ultrasound and an automated immunoassay (Elecsys^®^ AMH assay, Roche Diagnostics, Rotkreuz, Switzerland), respectively. Then, each BC patient and OD underwent a standard antagonist protocol. Briefly, ovarian stimulation was initiated either in the early follicular phase or randomly during the luteal phase [32]. Injections of recombinant follicle-stimulating hormone (FSH) were administered to BC patients and OD. From day 6 of stimulation, follicle growth was monitored using transvaginal ultrasound and oestradiol levels were measured in the serum, allowing the daily dose of FSH to be adjusted in order to maximise the response and minimise the risk of developing OHSS. A GnRH antagonist (Cetrotide^®^; Merck Serono, Fenil-sur-Corsier, Switzerland; Orgalutran^®^; Organon, Oss, The Netherlands; or Fyremadel^®^; Sun Pharmaceuticals, Hoofddorp, The Netherlands) was injected daily from day 6 of stimulation to prevent a premature LH surge. For BC patients with a HR+ tumour, an aromatase inhibitor (Letrozole; EG Labo, Brussels, Belgium) or a selective oestrogen receptor modulator (Tamoxifen; Eugia Pharma, Floriana, Malta) was also administered to reduce oestrogen production or action, respectively. Once three or more follicles had reached a diameter of at least 17 mm, ovulation and oocyte maturation were triggered by an injection of either recombinant human chorionic gonadotropin (Ovitrelle, Merck Serono, Fenil-sur-Corsier, Switzerland) and/or Decapeptyl (Ferring, Saint-Prex, Switzerland) depending on the estimated risk of OHSS and the anticipated degree of oocyte maturation. Approximately 35 h after triggering, the follicles were punctured vaginally under anaesthesia. The FFs and cumulus–oocyte complexes (COCs) were then collected. The CCs were then removed from each COC by enzymatic (Hyaluronidase, 80 IU/mL; Hyase, Vitrolife, Gothenburg, Sweden) and mechanical means, after which all the CCs from the same patient were pooled. The retrieved mature oocytes were vitrified for FP or oocyte donation (RapidVitTM, Vitrolife, Gothenburg, Sweden). The FFs and CCs were snap-frozen in liquid nitrogen and stored at −80 °C in the Germethèque Biobank until further use.

### 2.4. RNA Isolation, Reverse Transcription and Quantitative Polymerase Chain Reaction

Total RNA was isolated from CCs using RNeasy micro kits (Qiagen, Les Ulis, France), following the manufacturer’s instructions. The concentration, quality and integrity of each RNA sample were assessed using an automated microfluidic electrophoresis assay (High Sensitivity RNA ScreenTape Assay, Agilent Technologies, Les Ulis, France) on a 4200 TapeStation (Agilent Technologies, Les Ulis, France). Only samples with more than 40 ng of RNA were processed further. This did not appear to introduce a selection bias, as no significant differences were found in the number of COCs retrieved (*p* = 0.243) or the oocyte maturity rate (*p* = 0.692) between samples with RNA yields below and above the 40 ng threshold. Furthermore, the proportion of patients excluded from the cumulus cell analysis due to this post hoc eligibility criterion did not differ significantly between groups (*p* = 0.522).

For each RNA sample, a reverse transcription (RT) reaction was performed using 40 ng of total RNA on a T100 thermal cycler (BioRad, Marnes-la-Coquette, France). Briefly, a mixture of dNTP (0.5 mM, Thermo Fisher Scientific, France) and random hexamers (2.5 µM, Thermosfisher Scientific, France) was added to each extracted RNA sample, which was then incubated for 5 min at 65 °C. A second reaction mixture containing SuperScript IV Buffer (1×, Thermosfisher Scientific, Illkirch, France), DTT (5 mM, Thermo Fisher Scientific, Illkirch, France), SuperScript IV Reverse Transcriptase (200 U, Thermo Fisher Scientific, Illkirch, France) and RNaseOUT™ (40 U, Invitrogen, Villebon-sur-Yvette, France) was added to the previous RNA mixture. The samples were then incubated for 10 min at 23 °C, 10 min at 55 °C, and 10 min at 80 °C, after which they were stored at −20 °C until qPCR.

A qPCR mixture was prepared using Sybergreen Premix Ex TaqTM (Takara Bio, Inc., Kusatsu, Japan), RNase-free water (Thermo Fisher Scientific, Illkirch, France) and gene-specific primers (the forward and reverse primers for eleven genes that code for enzymes and regulators of cholesterol biosynthesis and for the internal standard control TATA box binding protein (TBP) are listed in Table S1; Eurogenetec, Seraing, Belgium, or Merck Serono, Fenil-sur-Corsier, Switzerland)). Then, 2 µL of 1:10 diluted cDNA was added to the mixture, and qPCR was performed on a LightCycler 480 (Roche Diagnosis, Mannheim, Germany) using the following program: an initial incubation at 95 °C for 10 min, followed by 40 cycles, each composed of a denaturation step (15 s at 95 °C), an annealing step (15 s at 59 °C) and an extension step (1 min at 72 °C). The results were analysed using LightCycler480 software 1.5.1. All gene expression levels were normalised to the level of the internal standard control TBP and analysed using the 2^−ΔΔCt^ method [33].

### 2.5. Quantification of Cholesterol and Its Intermediates in Follicular Fluids

**Figure 2:**
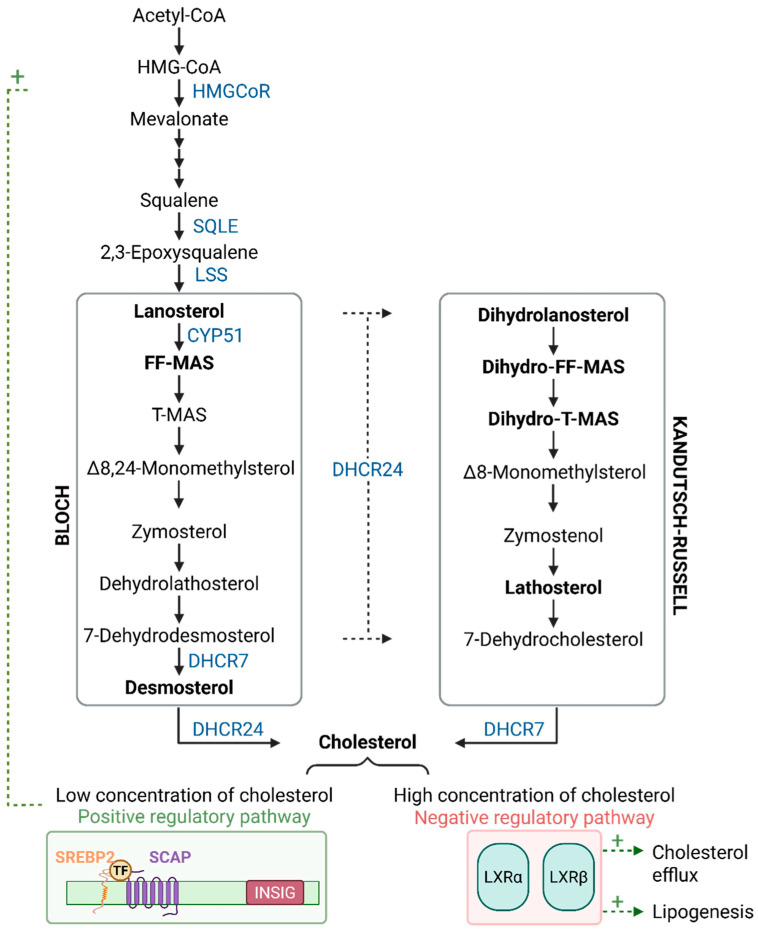
Cholesterol biosynthesis: the Bloch and Kandutsch–Russell pathways. All enzymes analysed in this study are shown in blue: 3-hydroxy-3methylglutaryl-coenzyme A reductase (HMGCoR), squalene epoxidase (SQLE), lanosterol synthase (LSS), lanosterol 14-α-demethylase (CYP51), 7-dehydrocholesterol reductase (DHCR7), and 24-dehydrocholesterol reductase (DHCR24). Intermediates measured from both pathways are depicted in bold: lanosterol, FF-MAS, and desmosterol for the Bloch pathway and dihydrolanosterol, dihydro-FF-MAS, dihydro-T-MAS, and lathosterol for the Kandutsch–Russell pathway. The cholesterol biosynthesis is finely controlled by regulatory mechanisms: a positive feedback loop (+) mediated by the SREBP2–SCAP complex (in green), which is activated when intracellular cholesterol levels decrease, and inhibitory controls exerted by INSIG and liver X receptors (LXRα and LXRβ, in red). Positive regulations are depicted by green dotted arrows. FF-MAS: follicular fluid–meiosis-activating sterol; INSIG: insulin induced-genes; SCAP: sterol regulatory element-binding protein cleavage-activating protein; SREBP2: sterol regulatory element-binding protein-2; T-MAS: testis-meiosis-activating sterol.

Sterols and oxysterols from the Bloch and Kandutsch–Russell pathways (see Figure 2) were quantified in 100 µL of FF. After alkaline hydrolysis, the sterols and oxysterols were extracted with cyclohexane and silylated. The O-trimethylsilylated sterol and di-trimethylsilylated oxysterol ethers were separated by gas chromatography (GC) from the lipid extracts following the methods outlined in a previous article [34]. Cholesterol was detected by flame ionisation detection using 5α-cholestane as an internal standard (ISTD). The non-cholesterol sterols (epicoprostanol, ISTD) and the oxysterols (2Hx-oxysterols, ISTD) were detected by a highly specific, sensitive mass spectrometer in selected ion monitoring mode (MS-SIM). Gas chromatographic separation and detection of cholesterol and 5α-cholestane (ISTD) were performed on a DB-XLB column with a 30 m × 0.25 mm i.d. × 0.25 µm film (J&W Scientific Alltech, Folsom, CA, USA) in a Hewlett-Packard (HP) 6890 series GC system (Agilent Technologies, Palo Alto, CA, USA), equipped with a flame ionisation detector (FID). Non-cholesterol sterols were separated on another DB-XLB column with a 30 m × 0.25 mm i.d. × 0.25 µm film (J&W Scientific Alltech, Folsom, CA, USA) in an HP 6890N Network GC system (Agilent Technologies, Waldbronn, Germany), connected with a direct capillary inlet system to an inert quadrupole mass selective detector HP5975B (Agilent Technologies, Waldbronn, Germany). For mass selective detection, electron-impact ionisation was applied with 70 eV. MS-SIM was performed by cycling the quadrupole mass filter between different *m*/*z* values at a rate of 3.7 cycles/s. The TMSi derivatives of the non-cholesterol sterols and the di-TMSi-derivatives of the oxysterols were monitored using the following masses: FF-MAS at *m*/*z* 482 (M+); 24.25-dihydro-FF-MAS at *m*/*z* 484 (M+); 24.25-dihydro-T-MAS at *m*/*z* 486; lathosterol at *m*/*z* 458 (M+); desmosterol at *m*/*z* 441 (M+ -15, M+ -CH3); lanosterol at *m*/*z* 393 (M+ -90-15, M+ -OTMSi-CH3); 22.23-dihydrolanosterol at *m*/*z* 395 (M+ -90-15, M+ -OTMSi-CH3). Peak integration was performed manually. Cholesterol was directly quantified by multiplying the ratios of the area under the curve of cholesterol or cholestanol to 5α-cholestane by 50 µg (ISTD amount). Non-cholesterol sterols and oxysterols were quantified using the ratios of the areas under the curve of the respective non-cholesterol sterols/oxysterols after MS-SIM analyses against internal standards, which were performed using standard curves for the listed sterols/oxysterols.

### 2.6. Statistical Analysis

Continuous data were presented as means with standard deviations depending on the distribution. The Shapiro–Wilk test was used to assess the normality of variable distributions. Comparisons between independent groups were performed using analysis of variance (ANOVA) or the Kruskal–Wallis test when the assumptions to apply ANOVA were not met. When appropriate (omnibus *p*-value less than 0.05), a post hoc test for two-by-two multiple comparisons was applied: Dunnett after ANOVA and Dunn after Kruskal–Wallis. In addition, multivariate analyses were conducted for comparisons with donors to take into account possible confounders: age and BMI, and AMH levels when appropriate. More precisely, multiple linear regression was performed. The normality of residuals was analysed as aforementioned. When necessary, a transformation (i.e., logarithmic) of the dependent variable was used. All statistical analyses were conducted using Stata version 15 (StataCorp, College Station, TX, USA). Two-tailed tests were applied, with the significance threshold set at *p* < 0.05. In addition to the post hoc multiple comparison procedures described above, a Sidak correction was applied in multivariate analyses comparing groups to control for type I error. No additional global correction was performed across the multiple biomarkers analysed, as these were not considered a single statistical family, and the analyses were exploratory. Effect sizes were systematically calculated and interpreted according to the recommendations of Cohen (0.2 = small, 0.5 = moderate, 0.8 = large).

## 3. Results

### 3.1. Ovarian Reserve Parameters and Response to Hormonal Stimulation

To infer the possible impact of BC on ovarian function, we compared the ovarian response to stimulation in thirty-six BC women (BC group) with that in sixty-four cancer-free women (OD group) (see Figure 1 and Table 1). There was no significant difference in age (OD: 32.4 ± 3.5 years vs. BC: 32.4 ± 3.9 years; *p* = 0.94) or BMI (OD: 23.4 ± 4.2 kg/m^2^ vs. BC: 23.5 ± 4.4 kg/m^2^; *p* = 0.91) between the BC and OD groups (Table 1). Comparisons between the OD group and the different BC molecular subgroups also showed no significant differences in age (OD vs. TN: 31.5 ± 3.6 years, *p* = 0.82; HR+: 33.3 ± 4.0 years, *p* = 0.93; HER2+: 32.8 ± 3.1 years, *p* = 0.98) or BMI (OD vs. TN: 23.1 ± 4.2 kg/m^2^, *p* > 0.99; HR+: 22.3 ± 5.4 kg/m^2^, *p* = 0.95; HER2+: 24.5 ± 4.3 kg/m^2^, *p* = 0.82), ensuring group comparability. Nevertheless, as age and BMI are well-known major determinants of female fertility, all subsequent statistical analyses were adjusted for these variables in order to limit potential confounding effects [35]. Regarding the ovarian reserve proxy, the women with BC displayed lower AMH levels than those in the OD group (2.8 ng/mL ± 1.7 vs. 3.9 ng/mL ± 2.3, *p* = 0.035). This was particularly evident in the TN subgroup (2.6 ± 1.4 ng/mL vs. 3.9 ± 2.3 ng/mL, *p* = 0.028), though not in the HR+ and HER2+ subgroups (OD vs. HR+: 3.2 ± 1.6 ng/mL, *p* = 0.77; HER2+: 2.9 ± 2.2 ng/mL, *p* = 0.16). AFC was similar between the OD and BC patient groups (22.8 ± 9.5 vs. 21.2 ± 9.3, *p* = 0.31). Similarly, no difference in AFC was observed between the OD group and each BC subgroup (OD vs. TN: 19.7 ± 9.3, *p* = 0.13; HR+: 23.0 ± 8.8, *p* = 0.76; HER2+: 21.8 ± 10.0, *p* = 0.63).

Regarding ovarian stimulation, a marginal increase in the total dose of FSH administered was observed between the OD and BC groups (2068 ± 721 IU vs. 2353 ± 965 IU, *p* = 0.10; Table 1). When BC was categorised according to its molecular subtype, only patients with TNBC required a significantly higher total dose of FSH compared to the OD group (2522 ± 865 IU vs. 2068 ± 721 IU; *p* = 0.017). Notably, the number of retrieved oocytes was statistically lower in the BC group than in the OD group (9.17 ± 5.7 vs. 13.4 ± 6.4; *p* < 0.001). Fewer oocytes were retrieved in all BC subgroups compared to the OD group (OD: 13.4 ± 6.4 vs. TN: 9.07 ± 6.6, *p* = 0.001; HR+: 8.00 ± 2.3, *p* = 0.036; HER2+: 9.86 ± 6.1, *p* = 0.023). This difference remained significant after adjusting for AMH levels (OD: 13.4 ± 6.4 vs. BC: 9.17 ± 5.7, *p* = 0.001; TN: 9.07 ± 6.6, *p* = 0.011; HR+: 8.00 ± 2.3, *p* = 0.022; HER2+: 9.86 ± 6.1, *p* = 0.053), suggesting that it is not due to the observed alteration of the ovarian reserve. Furthermore, a lower percentage of mature oocytes was obtained in the BC group compared with the OD group (69.6 ± 27.5% vs. 79.9 ± 14.7%, *p* = 0.002), with the difference being particularly noticeable in the HER2+ subgroup (61.5 ± 25.1% vs. 79.9 ± 14.7%, *p* = 0.001). The TN subgroup showed only a non-significant trend towards a reduction in oocyte maturity (73.8 ± 30.1% vs. 79.9 ± 14.7%, *p* = 0.076). A significant decrease in the percentage of atretic oocytes was observed in the BC group compared to the OD group (*p* = 0.044), whereas no significant differences were found between the OD and any of the BC subgroups.

### 3.2. Ovarian Response to Hormonal Stimulation According to Tumour Characteristics

Subgroup analyses were performed to assess the potential impact of tumour grade and dissemination on the ovarian response to stimulation (Tables S2 and S3). AMH serum levels were significantly lower in the Grade 3 subgroup than in both the OD group and the Grade 2 subgroup (Grade 3: 2.2 ± 1.2 ng/mL vs. OD: 3.9 ± 2.3 ng/mL, *p* = 0.002; Grade 3 vs. Grade 2: 3.7 ± 2.1 ng/mL, *p* = 0.011). With regard to ovarian stimulation parameters, the total dose of FSH tended to be higher in the Grade 3 subgroup than in the OD group (2488 ± 1197 IU vs. 2068 ± 720.9 IU, *p* = 0.06). In terms of stimulation outcomes, both the Grade 2 and Grade 3 subgroups had fewer retrieved oocytes than the OD group. However, no difference was observed between the two grade subgroups. Only the Grade 3 subgroup showed a significant decrease in the maturity rate compared to the OD group (OD: 79.9 ± 14.7% vs. Grade 2: 73.4 ± 23.2%, *p* = 0.22; OD vs. Grade 3: 69.3 ± 31.9%, *p* = 0.005). In terms of tumour dissemination, no significant differences were observed in terms of ovarian reserve (AMH and AFC) or ovarian response parameters (total FSH dose) between the localised and disseminated subgroups (Table S3). However, patients with disseminated tumours tended to have lower AMH blood levels than those in the OD group (2.6 ± 1.4 ng/mL vs. 3.9 ± 2.3 ng/mL, *p* = 0.091). In terms of ovarian stimulation outcomes, a significant decrease in the oocyte maturity rate was observed in the disseminated subgroup compared to the OD group (69.7 ± 26.9% vs. 79.9 ± 14.7%, *p* = 0.023), whereas only a marginal difference was seen for the localised subgroup (72.8 ± 30.1% vs. 79.9 ± 14.7%, *p* = 0.085). Finally, no significant differences were found between patients with or without BRCA1 mutation regarding the total dose of FSH administered, the number of retrieved oocytes, oocyte maturity and atretic rates (see Table S4).

### 3.3. Cholesterol Homeostasis in Cumulus Cells and Follicular Fluids According to the Different Molecular Subtypes of Breast Cancer

#### 3.3.1. Triple-Negative Breast Cancer

**Figure 3:**
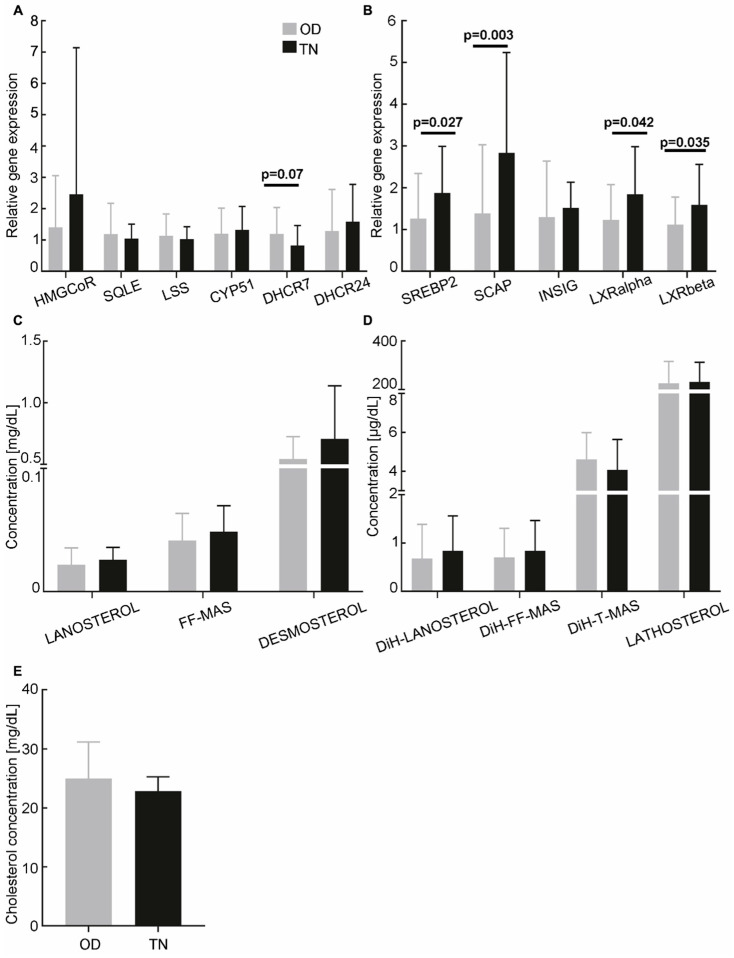
Cholesterol biosynthesis in triple-negative breast cancer (TNBC) patients. (**A**,**B**) Relative expression levels of cholesterol biosynthesis enzymes and regulators in cumulus cells from TNBC patients (*n* = 12, except DHCR7: *n* = 11; black) and oocyte donors (OD; *n* = 55, except HMGCoR: *n* = 54; grey). The RT-qPCR data were normalised using the TBP housekeeping gene. (**C**,**D**) Concentrations of cholesterol biosynthesis intermediates in follicular fluids from the Bloch (**C**) and Kandutsch–Russell (**D**) pathways (OD, *n* = 55; TNBC, *n* = 12). (**E**) Concentration of cholesterol in follicular fluids from OD (*n* = 55) and TNBC (*n* = 12). Statistical analyses were performed using ANOVA or the Kruskal–Wallis test when assumptions for ANOVA were not met, and multivariate analyses adjusting for age and BMI. HMGCR: 3-hydroxy-3-methylglutaryl-coenzyme A reductase; SQLE: squalene epoxidase; LSS: lanosterol synthase; CYP51: lanosterol 14-α-demethylase; DHCR7: 7-dehydrocholesterol reductase; DHCR24: 24-dehydrocholesterol reductase; SREBP2: sterol regulatory element-binding protein-2; SCAP: sterol regulatory element-binding protein cleavage-activating protein; INSIG: insulin-induced genes; DiH: dihydro.

As reported in Figure 3A, there were no significant differences in the expression of genes coding for cholesterol biosynthesis enzymes in CCs from TNBC patients compared to OD; only the expression level of DHCR7 was marginally reduced (HMGCoR: 2.46 ± 4.68 vs. 1.40 ± 1.65, *p* = 0.33; SQLE: 1.04 ± 0.46 vs. 1.19 ± 0.98, *p* = 0.63; LSS: 1.0 ± 0.40 vs. 1.13 ± 0.70, *p* = 0.66; CYP51: 1.32 ± 0.75 vs. 1.20 ± 0.82, *p* = 0.54; DHCR7: 0.83 ± 0.64 vs. 1.1 ± 0.85, *p* = 0.07; DHCR24: 1.59 ± 1.19 vs. 1.28 ± 1.33, *p* = 0.26). The expression levels of the positive regulators SREBP2 and SCAP were significantly higher in the TNBC subgroup than in the OD group (SREBP2: 1.88 ± 1.12 vs. 1.26 ± 1.08, *p* = 0.027; SCAP: 2.83 ± 2.40 vs. 1.38 ± 1.64, *p* = 0.003; Figure 3B). Among the negative regulators, LXRα and LXRβ expression levels were also significantly increased (LXRα: 1.84 ± 1.14 vs. 1.23 ± 0.85, *p* = 0.042; LXRβ: 1.59 ± 0.97 vs. 1.12 ± 0.66, *p* = 0.035), while no difference was observed in INSIG expression levels (1.52 ± 0.62 vs. 1.30 ± 1.34; *p* = 0.23). Low-to-moderate effect sizes were observed for all of these significantly different parameters (see Table S5). Regarding the levels of cholesterol and its intermediates in FF, no differences were observed between TNBC patients and OD. Overall, our data show that, despite a modulation of the regulatory pathways of cholesterol biosynthesis in TNBC, there is no impact on the expression levels of the cholesterol biosynthesis enzymes in CC, nor on the levels of cholesterol and its intermediates in FF (Figure 3C–E).

#### 3.3.2. Hormone-Receptor Positive Breast Cancer

**Figure 4:**
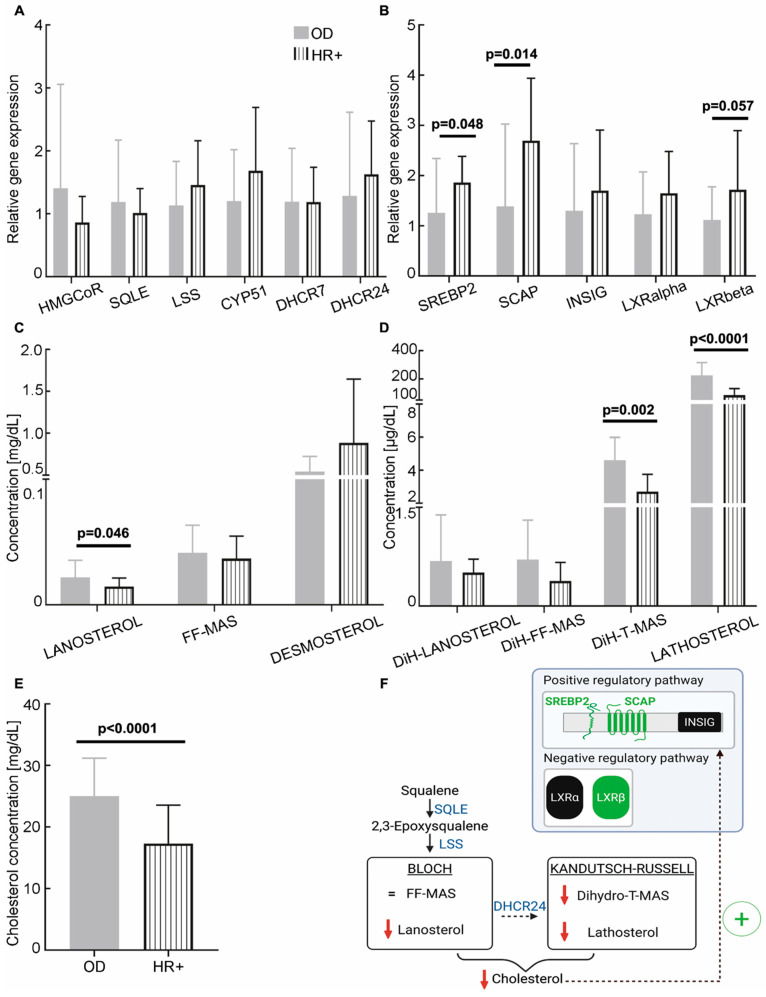
Cholesterol biosynthesis in hormone-dependent breast cancer (HR+) patients. (**A**,**B**) Relative expression levels of cholesterol biosynthesis enzymes and regulators in cumulus cells from HR+ patients (*n* = 6; vertical hatch) and oocyte donors (OD; *n* = 55, except HMGCoR: *n* = 54; grey). The RT-qPCR data were normalised using the TBP housekeeping gene. (**C**,**D**) Concentrations of cholesterol biosynthesis intermediates in follicular fluids from the Bloch (**C**) and Kandutsch–Russell (**D**) pathways (OD, *n* = 55; HR+, *n* = 6). (**E**) Concentration of cholesterol in follicular fluids from OD (*n* = 55) and HR+ patients (*n* = 6). (**F**) Schematic model summarising the observed dysregulations in HR+ patients. Red downward arrows indicate a decrease in concentration. A black dotted arrow with a green (+) indicates positive putative regulations. Statistical analyses were performed using ANOVA or the Kruskal–Wallis test when the assumptions for ANOVA were not met, and multivariate analyses adjusting for age and BMI. HMGCoR: 3-hydroxy-3-methylglutaryl-coenzyme A reductase; SQLE: squalene epoxidase; LSS: lanosterol synthase; CYP51: lanosterol 14-α-demethylase; DHCR7: 7-dehydrocholesterol reductase; DHCR24: 24-dehydrocholesterol reductase; SREBP2: sterol regulatory element-binding protein-2; SCAP: sterol regulatory element-binding protein cleavage-activating protein; INSIG: insulin-induced genes; DiH: dihydro.

There was no significant difference in the expression of genes coding for cholesterol biosynthesis enzymes in HR+ BC patients’ CCs compared to OD (HMGCoR: 0.86 ± 0.41 vs. 1.40 ± 1.65, *p* = 0.46; SQLE: 1.01 ± 0.39 vs. 1.19 ± 0.98, *p* = 0.83; LSS: 1.46 ± 0.70 vs. 1.13 ± 0.70, *p* = 0.17; CYP51: 1.68 ± 1.00 vs. 1.20 ± 0.82, *p* = 0.15; DHCR7: 1.19 ± 0.55 vs. 1.19 ± 0.85, *p* = 0.83; DHCR24: 1.63 ± 0.85 vs. 1.28 ± 1.33, *p* = 0.24; see Figure 4A). However, SREBP2 and SCAP expression levels were significantly higher in the HR+ subgroup than in the OD group (1.86 ± 0.52 vs. 1.26 ± 1.08, *p* = 0.048; 2.70 ± 1.24 vs. 1.38 ± 1.64, *p* = 0.014, respectively; Figure 4B). In addition, LXRβ expression levels tended to be higher in the HR+ sub-group than in the OD group (1.72 ± 1.18 vs. 1.12 ± 0.66, *p* = 0.057). No difference was observed in the expression of INSIG (1.70 ± 1.21 vs. 1.30 ± 1.34, *p* = 0.27) or LXRα (1.65 ± 0.83 vs. 1.12 ± 0.66, *p* = 0.20). In the FF, three compounds were found to be significantly reduced in HR+ BC patients compared to OD: lanosterol (14.6 ± 6.76 µg/dL vs. 21.8 ± 13.7 µg/dL; *p* = 0.046), dihydro-T-MAS (2.71 ± 1.05 µg/dL vs. 4.61 ± 1.38 µg/dL; *p* = 0.002) and lathosterol (0.09 ± 0.05 mg/dL vs. 0.23 ± 0.09 mg/dL; *p* < 0.0001; see Figure 4C,D). Finally, the cholesterol concentration was significantly reduced in the FF of HR+ BC patients compared to OD (17.3 ± 6.22 mg/dL vs. 25.0 ± 6.18 mg/dL, *p* < 0.0001; Figure 4E). Low-to-moderate effect sizes were observed for all of the significantly different parameters (see Table S5). Overall, our results indicate that, although there is no impact on the expression levels of enzymes of the cholesterol biosynthesis pathway, there is a clear decrease in cholesterol and its intermediate levels in HR+ BC patients (Figure 4F).

#### 3.3.3. HER2-Positive Breast Cancer

**Figure 5:**
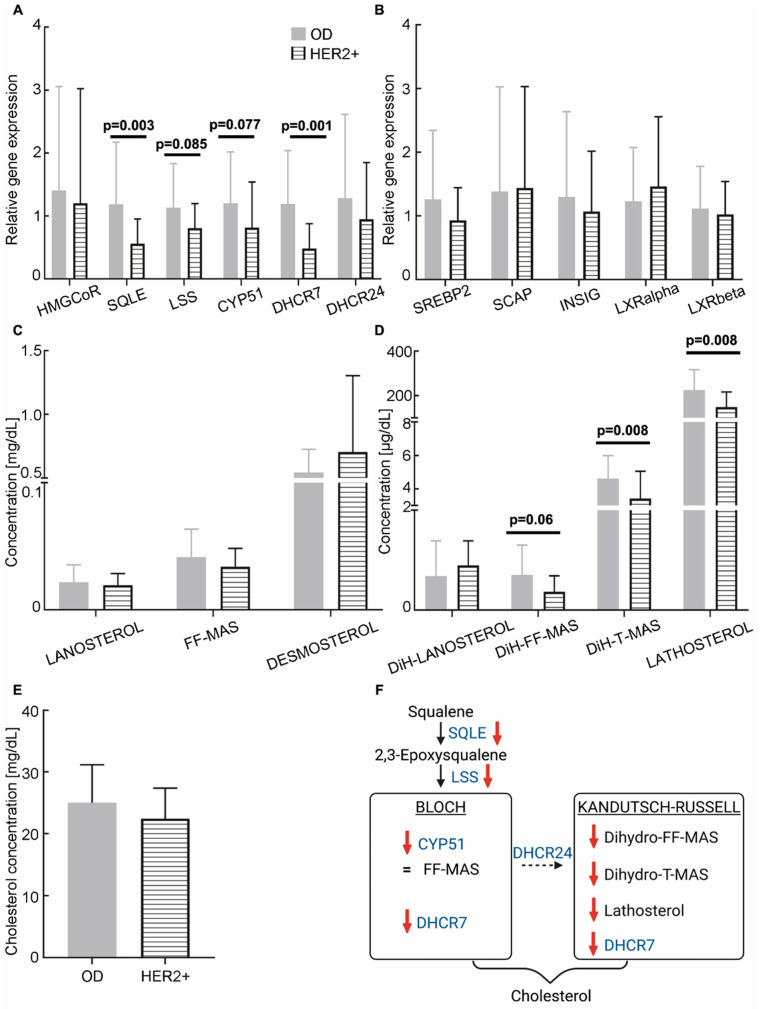
Cholesterol biosynthesis in HER2-overexpressing breast cancer (HER2+) patients. (**A**,**B**) Relative expression levels of cholesterol biosynthesis enzymes and regulators in cumulus cells from HER2+ patients (*n* = 10; horizontal hatch) and oocyte donors (OD; *n* = 55, except HMGCoR: *n* = 54; grey). The RT-qPCR data were normalised using the TBP housekeeping gene. (**C**,**D**) Concentrations of cholesterol biosynthesis intermediates in follicular fluids from the Bloch (**C**) and Kandutsch–Russell (**D**) pathways (OD, *n* = 55; HR+, *n* = 10). (**E**) Cholesterol concentration in follicular fluids from OD (*n* = 55) and HER2+ patients (*n* = 10). (**F**) Schematic model summarising the observed dysregulations in HER2+ patients. Red downward arrows indicate a decrease in concentration. Statistical analyses were performed using ANOVA or the Kruskal–Wallis test when the assumptions for ANOVA were not met, and multivariate analyses adjusting for age and BMI. HMGCoR: 3-hydroxy-3-methylglutaryl-coenzyme A reductase; SQLE: squalene epoxidase; LSS: lanosterol synthase; CYP51: lanosterol 14-α-demethylase; DHCR7: 7-dehydrocholesterol reductase; DHCR24: 24-dehydrocholesterol reductase; SREBP2: sterol regulatory element-binding protein-2; SCAP: sterol regulatory element-binding protein cleavage-activating protein; INSIG: insulin-induced genes; DiH: dihydro.

No significant differences were observed in the expression of the HMGCoR and DHCR24 genes in CCs from HER2+ BC patients compared to OD (1.20 ± 1.82 vs. 1.40 ± 1.65; *p* = 0.42 and 0.95 ± 0.90 vs. 1.28 ± 1.33; *p* = 0.24, respectively), whereas SQLE and DHCR7 expressions were significantly decreased (0.56 ± 0.39 vs. 1.19 ± 0.98, *p* = 0.003; 0.48 ± 0.39 vs. 1.19 ± 0.85, *p* = 0.001, respectively; see Figure 5A). In addition, there was a marginal reduction in LSS and CYP51 expression (0.81 ± 0.40 vs. 1.13 ± 0.70, *p* = 0.085; 0.82 ± 0.73 vs. 1.20 ± 0.82, *p* = 0.077, respectively). No significant difference in the expression of regulatory genes was observed between women with HER2+ BC and OD (Figure 5B). Regarding the concentration of cholesterol intermediates in FF, a reduction was observed in the concentration of dihydro-FF-MAS (0.37 ± 0.32 µg/dL vs. 0.71 ± 0.60 µg/dL; *p* = 0.06), dihydro-T-MAS (3.42 ± 1.64 µg/dL vs. 4.61 ± 1.38 µg/dL; *p* = 0.008) and lathosterol (0.15 ± 0.07 mg/dL vs. 0.23 ± 0.09 mg/dL; *p* = 0.008) in HER2+ BC patients compared to ODs (Figure 5C,D). There was no significant difference in cholesterol concentration between the HER2+ subgroup and the OD group (22.4 ± 4.97 mg/dL vs. 25.0 ± 6.18 mg/dL; *p* = 0.27; see Figure 5E). Moderate effect sizes were observed for all of the significantly different parameters (see Table S5). Taken together, our data show that the levels of enzymes involved in cholesterol synthesis, as well as cholesterol intermediates, are reduced in CCs and FF of HER2+ BC patients (Figure 5F).

## 4. Discussion

This case-control study shows that BC itself appears to affect ovarian function and cholesterol homeostasis in CC and/or FF in a tumour-subtype-dependent manner. Compared to healthy women (OD), BC patients showed a reduced ovarian response to stimulation and a modulation of gene expression levels of enzymes and regulators of cholesterol biosynthesis in CC. Consistently, GC-FID and GC-MS-SIM analyses revealed altered concentrations of cholesterol and its intermediates in FF from BC patients compared to OD. Together, these findings provide mechanistic insights into the potential detrimental effect of cancer on ovarian function and suggest that tumour-driven metabolic disturbances may compromise oocyte competence. These original results are important because they may have a potential impact on the fertility restoration of these women.

Our study highlights a decrease in serum AMH levels in BC patients compared to OD, which is consistent with the findings of previous studies [6,36]. Conversely, we found no difference in AFC between the two study groups, in line with studies reporting preserved ovarian reserve in BC patients [37,38,39]. Importantly, as AMH exhibits minimal intra-cycle fluctuations [40,41], it is often considered a more reliable marker of ovarian reserve than AFC. This is particularly pertinent given that our FP protocols were initiated at random starts of the cycle, thus supporting the idea that ovarian reserve is altered in BC patients compared with controls. Interestingly, our study found that TNBC patients had lower AMH levels than OD, in contrast to HR+ and HER2+ patients. This may explain why higher gonadotropin doses were administered to TNBC patients, in order to maximise the ovarian response to stimulation. However, previous studies have found no differences in ovarian reserve parameters specific to BC molecular subtypes [12,42]. These discrepancies could be due to differences in methodology, such as the use of different assays and the lack of standardised measurement protocols [43]. Other confounding factors may also be at play, including patient age and BMI, oral contraceptive use, the phase of the menstrual cycle, and heterogeneity in study design (e.g., differences in the choice of control group) [35]. Regarding FP outcomes, our data revealed poorer ovarian responses to stimulation in BC patients than in OD, notably characterised by a lower number of oocytes retrieved. Importantly, this difference remained significant after adjustment for AMH levels, suggesting that it is not solely explained by alterations in ovarian reserve. These findings are consistent with previous studies and meta-analyses conducted in cancer patients [5,6,10]. In our cohort, HR+ patients had the lowest number of retrieved oocytes, while HER2+ patients had the lowest oocyte maturity rate, which is consistent with previous studies showing a differential response to ovarian stimulation among BC molecular subtypes [10,12,44]. However, previous studies have not identified any significant differences in the number or maturity of retrieved oocytes between cancer and non-cancer populations [15,38,45], or between BC molecular subtypes [42]. The discrepancies between BC subgroups may be linked to their constitution: TNBC vs. non-TNBC in [12], ER+ vs. ER− in [46], TNBC vs. all HR+ (including HER2+) in [47], and TNBC/HR+/HER2+ (HR+) in our study.

We further investigated whether tumour characteristics could affect ovarian function. We observed a decrease in AMH levels from Grade 2 to Grade 3 BC, suggesting that greater tumour aggressiveness may be linked with a diminished ovarian reserve. Tumour invasion also appears to be an important factor, given that oocyte maturation rates were significantly lower in patients with disseminated cancer than in the OD group. Previous studies have already reported an adverse impact of tumour grade, but not stage, on ovarian response [13]. However, others have found no impact of either grade or stage [48]. Overall, our findings, alongside those from the literature, suggest that an increase in tumour burden and systemic spread may progressively compromise ovarian function. Although previous studies have associated BRCA1 mutations with impaired ovarian function and premature ovarian failure [8,14,49,50,51], we observed no significant differences in ovarian reserve or oocyte yield between carriers and non-carriers of BRCA1 mutations. This finding is consistent with the results of recent studies [52,53,54,55], but differs from previous reports describing a lower number of mature oocytes in BRCA1 carriers compared with non-carriers [8,56]. Several methodological differences may explain these discrepancies, such as the characteristics of the cohorts. For example, the upper age limit for women can be up to 40 years old or even older [8,49,52,54,55,56], which can bias the ovarian response, particularly since not all studies perform multivariate analyses adjusted for age [14,49,50,52,56]. Additionally, some studies focus on the impact of BRCA mutations in a non-oncological context [54,55], while others do not discriminate between BRCA1 and BRCA2 [14,50,52,56] or mention BC molecular subtypes [14,51,53,54,55,56]. There is also substantial variability in terms of ovarian stimulation protocols and the administration of letrozole or tamoxifen. These factors may therefore partly explain the inconsistent findings regarding BRCA status. Future research is required to determine whether BRCA mutations may affect the ovarian response to stimulation and to investigate other genes linked to hereditary BC, such as ATM and TP53 [57].

The mechanisms underlying the potential detrimental impact of cancer on ovarian function are not well understood. To address this issue, we examined two key indicators of the follicular microenvironment that are discarded during oocyte retrieval in clinical practice: CC and FF. Their molecular and metabolic profiles reflect the functional status of the oocyte and provide valuable insights into the determinants of oocyte competence in both physiological and pathological contexts [58,59]. For this analysis, we adopted a targeted approach focusing on cholesterol biosynthesis, as this process is essential for female fertility. Indeed, cholesterol is a key component of cell membranes and a precursor of steroidogenesis, which occurs in theca and granulosa cells [60]. Notably, FF-MAS, an intermediate of cholesterol biosynthesis, has been demonstrated to accumulate in the ovary and to induce meiotic resumption during in vitro maturation [20,21,22,23,24,61]. Furthermore, cholesterol itself appears to play a pivotal role in female fertility, as experimental data indicate that both cholesterol depletion [62] and excess [28] impair oocyte activation and developmental competence. This highlights the importance of precise regulation of cholesterol homeostasis for ovarian function.

Our analyses showed that HER2+ patients seem to exhibit concomitant downregulation of several cholesterol biosynthesis enzymes (SQLE, DHCR7, CYP51) in CC, and a decrease in multiple Kandutsch–Russell pathway intermediates in FF. In this context, the downregulation of CYP51 may be relevant because this enzyme catalyses FF-MAS synthesis, and FF-MAS is crucial for oocyte maturation. These findings support the hypothesis that HER2+ BC may interfere with follicular growth and the competence of oocytes to mature. This would explain the lower number of retrieved and mature oocytes in this subgroup. In patients with HR+ BC, we observed a potential decrease in the concentrations of several intermediates and cholesterol in FF, as well as transcriptional activation of SREBP2-SCAP. The latter may be a compensatory response to sterol depletion. Altered cholesterol concentrations may impair steroidogenesis, which is essential for follicle growth and oocyte competence [63]. Together with altered lipid composition in the follicular environment [64], this alteration could explain the lower number of oocytes retrieved in HR+ patients. Overall, our data suggest that dysregulation in cholesterol biosynthesis may underlie the altered ovarian function in HER2+ and HR+ BC patients, at least in part. TNBC appeared to be associated with increased expression of positive (SREBP2 and SCAP) and negative (LXRα and LXRβ) regulators of cholesterol biosynthesis, with no modulation of enzyme expression in CC or sterol levels in FF observed. Although diminished ovarian reserve has been linked to reduced cholesterol metabolism in granulosa cells [65], our data suggest that the lower AMH levels observed in TNBC patients are unlikely to stem from altered cholesterol biosynthesis. This emphasises the importance of following an integrative approach that combines transcriptomics, metabolomics, and clinical outcomes in order to better characterise how tumour-related disturbances affect ovarian function. For instance, BC is known to induce systemic inflammation and oxidative stress [66], which can disrupt follicular steroidogenesis and the survival of primordial follicles, as demonstrated in mouse models [67]. Furthermore, elevated cytokine levels in the follicular microenvironment have been associated with a reduced ovarian response to stimulation [68]. It would therefore be relevant to further investigate additional pathways, such as inflammatory and oxidative stress signalling, to gain deeper insights into the pathophysiological mechanisms through which BC could alter ovarian function.

Our study presents some limitations. First, we selected oocyte donors as a control group because they are a selected population with no apparent deregulation of ovarian function. However, as this is an ideal population, it may introduce a bias into our analysis, as the observed alterations in cancer patients may be due to inherent infertility rather than to the cancer itself. Nevertheless, selecting subfertile or infertile patients undergoing ART procedures as a control group could also introduce bias, particularly given that infertility is often multifactorial. Second, the subgroup analyses of cancer molecular subtypes, grade, nodal status, and BRCA are based on small sample sizes, particularly in the HR+ subgroup. This is because several HR+ patients were excluded from our study as they had undergone breast cancer surgery before fertility preservation. To mitigate the risk of overinterpreting multiple comparisons, we complemented *p*-value-based inference with the systematic reporting of effect sizes and their confidence intervals. Several moderate-to-large effects were observed across biologically related biomarkers, strengthening the consistency and biological plausibility of the findings despite the absence of a global multiplicity correction. Nevertheless, further large-scale studies are necessary to confirm our findings. In addition, as most HER2+ and HR+ BC patients received letrozole or tamoxifen during the stimulation protocol, the observed effects on ovarian stimulation outcomes and alterations to cholesterol homeostasis in the follicular environment may be due to this supplementation rather than to the cancer itself. Further study is therefore required to distinguish between these two factors.

Our study provides clinically relevant insights for the management of BC patients undergoing FP. Our findings indicate that ovarian reserve and response to stimulation vary according to BC molecular subtype. This information could be used to tailor fertility FP strategies and counselling for each patient. Typically, 10 to 15 mature oocytes are needed to hope for a successful live birth [69]. In our cohort, however, women diagnosed with BC had, on average, fewer than 10 oocytes retrieved. Importantly, our findings and the existing literature suggest that oocyte functionality may be impaired in BC patients. Therefore, a higher number of oocytes may be required in this population. In this context, strategies to accumulate oocytes could be particularly relevant. However, implementing these strategies can be challenging due to the urgency of initiating chemotherapy or surgery. An alternative approach could be to consider oocyte accumulation after remission, if ovarian reserve permits, in order to optimise the chances of success. This approach is supported by growing evidence that oocyte cryopreservation is oncologically safe, with no detrimental effects on disease-free survival, recurrence, or overall survival [70]. Importantly, it is now clearly reported that pregnancy after BC is safe for these women [71]. Furthermore, for patients with the HER2+ subtype, who seemed to exhibit the lowest oocyte maturation rates in our cohort, experimental strategies could be considered to increase the number of competent oocytes. These patients appeared to exhibit a downregulation of multiple genes and intermediates in the cholesterol biosynthesis pathway compared with OD. In this context, in vitro maturation (IVM) rescue using medium supplemented with FF-MAS, as previously demonstrated by Torkashvand et al. [24], could enhance both nuclear and cytoplasmic maturation. Such targeted modulation in IVM medium may help increase the yield of competent oocytes in HER2+ patients, providing a potential approach to overcome subtype-specific deficits in oocyte maturation.

## 5. Conclusions

In conclusion, our study suggests that BC itself may cause ovarian dysfunction, in a subtype-specific manner, potentially via dysregulation of cholesterol biosynthesis, which could compromise the ability of oocytes to mature. Our findings emphasise the importance of deciphering the multifactorial mechanisms linking cancer and ovarian dysfunction using an integrative, multi-omics approach. From a clinical perspective, our results support personalised FP strategies based on tumour subtype. They also highlight the need to identify robust predictive biomarkers of oocyte competence and fertility in cancer patients to implement personalised FP strategies.

## Figures and Tables

**Table 1:** Characteristics and ovarian stimulation parameters and response of breast cancer patients and oocyte donors, categorised by molecular subtype of breast cancer.

	OD (*n* = 64)	BC (*n* = 36)	TN (*n* = 15)	HR+ (*n* = 7)	HER2+ (*n* = 14)	*p*-Value
Patient characteristics						
Age (years)	32.4 ± 3.9	32.4 ± 3.5	31.5 ± 3.6	33.3 ± 4.0	32.8 ± 3.1	NS
BMI (kg/m^2^)	23.4 ± 4.2	23.5 ± 4.4	23.1 ± 4.2	22.3 ± 5.4	24.5 ± 4.3	NS
AMH (ng/mL)	(*n* = 43) 3.9 ± 2.3	**(*n* = 27)** **2.8 ± 1.7**	**(*n* = 11)** **2.6 ± 1.4**	(*n* = 6) 3.2 ± 1.6	(*n* = 10) 2.9 ± 2.2	**^1^ 0.035** **^2^ 0.028** ^3^ NS ^4^ NS
AFC	(*n* = 60) 22.8 ± 9.5	(*n* = 34) 21.2 ± 9.3	(*n* = 14) 19.7 ± 9.3	(*n* = 7) 23.0 ± 8.8	(*n* = 13) 21.8 ± 10	NS
Ovarian stimulation						
Total dose of FSH (IU)	2068 ± 721	* 2353 ± 965	**2522 ± 865**	2070 ± 699	2315 ± 1182	^1^* 0.10 **^2^ 0.017** ^3^ NS ^4^ NS
Administration of Tamoxifen Letrozole	0/64 0/64	(*n* = 34) 10/34 10/34	0/15 2/15	4/7 3/7	(*n* = 12) 6/12 5/12	NA
Ovarian response						
Retrieved oocytes	13.4 ± 6.4	**9.17 ± 5.7**	**9.07 ± 6.6**	**8.00 ± 2.3**	**9.86 ± 6.1**	**^1^ <0.001** **^2^ 0.001** **^3^ 0.036** **^4^ 0.023**
Oocyte maturity (%)	79.9 ± 14.7	**69.6 ± 27.5**	* 73.8 ± 30.1	76.5 ± 26.0	**61.5 ± 25.1**	**^1^ 0.002** ^2^* 0.076 ^3^ NS **^4^ 0.001**
Atretic oocytes (%)	8.13 ± 11.2	**4.30 ± 7.60**	5.09 ± 9.68	3.17 ± 5.42	4.02 ± 6.30	**^1^ 0.044** ^2^ NS ^3^ NS ^4^ NS

Data are presented as the mean ± standard deviation. Bold values indicate statistically significant results (*p* < 0.05), while asterisks (*) denote marginal differences (0.05 ≤ *p* ≤ 0.1). ^1^ Comparison OD vs. BC; ^2^ OD vs. TN; ^3^ OD vs. HR+; ^4^ OD vs. HER2+. Statistical analyses were performed using ANOVA or the Kruskal–Wallis test when the assumptions for ANOVA were not met. Multivariate analyses, adjusting for age and BMI, were applied for AMH, AFC, ovarian stimulation and ovarian response parameters in comparisons with OD controls. The percentage of mature oocytes was calculated as (number of mature oocytes/number of retrieved oocytes × 100), and the percentage of atretic oocytes as (number of atretic oocytes/number of retrieved oocytes × 100). AFC: antral follicle count; AMH: anti-Mullerian hormone; BC: breast cancer; BMI: body mass index; FSH: follicle-stimulating hormone; HER2+: human epidermal growth factor receptor-2 positive; HR+: hormone-receptor positive; NS: non-significant; NA: not applicable; OD: oocyte donor; TN: triple negative.

**Table 2:** Tumour characteristics of women with breast cancer.

	BC (*n* = 36)	TN (*n* = 15)	HR+ (*n* = 7)	HER2+ (*n* = 14)
BRCA1 mutation [*n* (%)]	7/33 (21%)	6/15 (40%)	1/7 (14%)	0/11 (0%)
Grade	(*n* = 33)	(*n* = 14)	(*n* = 6)	(*n* = 13)
1	0	0	0	0
2	15	3	5	7
3	18	11	1	6
Stade (TNM)	(*n* = 32)	(*n* = 14)	(*n* = 6)	(*n* = 12)
I	8	5	0	3
II	21	9	5	7
III	3	0	1	2
IV	0	0	0	0
Lymph node invasion	(*n* = 32)	(*n* = 14)	(*n* = 6)	(*n* = 12)
Yes	16	5	5	6
No	16	9	1	6

BC: Breast cancer; BRCA: breast cancer gene; HER2+: human epidermal growth factor receptor-2 positive; HR+: hormone-receptor positive; TN: triple negative; TNM grading: Tumour, Node, Metastasis.

## Data Availability

The data presented in this study are available on request from the corresponding author due to the sensitive nature of the underlying medical data.

## Supplementary Materials

The following supporting information can be downloaded at https://www.mdpi.com/article/10.3390/cancers18091451/s1, Table S1: Forward and reverse primer sequences used for real-time quantitative PCR (RT-qPCR); Table S2: Bio-clinical characteristics of patients and their ovarian response to stimulation according to tumour grade; Table S3: Bio-clinical characteristics of patients and ovarian response to stimulation according to lymph node invasion; Table S4: Bio-clinical characteristics of patients and ovarian response to stimulation according to BRCA gene mutation status; Table S5: *p*-Values and effect sizes for the comparative analysis of the relative expression levels of cholesterol biosynthesis enzymes and regulators, and cholesterol biosynthesis intermediate concentrations, between oocyte donors and breast cancer molecular subgroups.

## Abbreviations

The following abbreviations are used in this manuscript:

FP	Fertility preservation
FF-MAS	Follicular fluid meiosis activating sterol
CC	Cumulus cell
FF	Follicular fluid
ART	Assisted reproductive technology
BC	Breast cancer
OHSS	Ovarian hyperstimulation syndrome
OD	Oocyte donor
ER	Estrogen receptor
PR	Progesterone receptor
HER2+	Human epidermal growth factor receptor 2 positive
TN	Triple negative
TNM	Tumour, Node and Metastasis
BRCA1/2	Breast cancer 1/2
AFC	Antral follicle count
AMH	Anti-Müllerian hormone
FSH	Follicle-stimulating hormone
COC	Cumulus–oocyte complex
RT	Reverse transcriptase
GC	Gas chromatography
ISTD	Internal standard
MS-SIM	Mass spectrometer-selected ion monitoring mode
HP	Hewlett-Packard
FID	Flame ionisation detector
ANOVA	Analysis of variance
IVM	In vitro maturation
