# Supplementary material for: Impact of Breast Cancer on Ovarian Function: Dysregulation of Cholesterol Homeostasis in Cumulus Cells and Follicular Fluid
Source: Cancers (Basel). 2026 May 1;18(9):1451. doi: 10.3390/cancers18091451 (PMC13163015; doi:10.3390/cancers18091451)
Supplement: Supplementary file 1 [file cancers-18-01451-s001.zip › Table S1.pdf]

Table S1: Forward and reverse primer sequences used for real time quantitative PCR (RT-qPCR)

Gene	Forward Primers	Reverse Primers
**TBP**	CGGCTGTTTAACTTCGCTTC	CACACGCCAAGAAACAGTGA
**HMG-coA Reductase**	GATGGGAGGCCACAAAGAG	TTCGGTGGCCTCTAGTGAGA
**SQLE**	CTCTCAGGCCTGCCTTTCATT	CCAGCTCCCACGATGATAACT
**LSS**	GAGCGGCGTTATTTGCAGAG	CCCCAGCAATGTTTTCCTGC
**CYP51**	GTTTCAGACGCAGGGACAGA	TCAGTCAAAGGACGCCCATC
**DHCR7**	GGTGGGCGCAGGACTTTAG	CCCTTGAGATGCGGTTCTGT
**DHCR24**	CTGTCTCACTACGTGTCGGG	CATCAAGCTCAGGCAACACG
**SREBP2**	GAGACCATGGAGACCCTCAC	GGAGCTACACAGCTGTTCTGA
**SCAP**	TCCTCATCGGCTACTTCACC	GTTCAGGTCTGCTAGCTCCA
**INSIG**	TCACACTGGCTGCACTATCC	ACAGTTGCCAAGAAGGCAAT
**LXRα**	AGGAGTGTCGGCTTCGCAAA	CTCTTCTTGCCGCTTCAGTTT
**LXRβ**	TCATCGCCATCAACATCTTC	GAAGACCTGCTCCGAGTGC

TBP (housekeeping gene): TATA Box binding Protein, HMG-coA Reductase: Hydroxylmethylglutaryl coenzyme A reductase, SQLE: squalene epoxidase, LSS: lanosterol synthase, CYP51: lanosterol 14-alpha demethylase, DHCR7: 7-dehydrocholesterol reductase, DHCR24: 24-dehydrocholesterol reductase, SREBP2: Sterol Regulatory Element-Binding Protein-2, SCAP: Sterol regulatory element-binding protein cleavage-activating protein, INSIG: Insulin Induced-genes, LXRα and β: Liver X Receptors alfa and beta.
