# Supplementary material for: Impact of Breast Cancer on Ovarian Function: Dysregulation of Cholesterol Homeostasis in Cumulus Cells and Follicular Fluid
Source: Cancers (Basel). 2026 May 1;18(9):1451. doi: 10.3390/cancers18091451 (PMC13163015; doi:10.3390/cancers18091451)
Supplement: Supplementary file 1 [file cancers-18-01451-s001.zip › Table S2.pdf]

Table S2: Characteristics of patients and their ovarian response to stimulation according to tumour grade.

	OD (n = 64)	Grade 2 (n = 15)	Grade 3 (n = 18)	p-value
Patient characteristics				
Age (years)	32.4 ± 3.9	33.0 ± 3.4	31.7 ± 3.5	NS
BMI (kg/m²)	23.4 ± 4.2	23.8 ± 4.9	23.6 ± 4.4	NS
AMH (ng/mL)	(n = 43)<br>3.9 ± 2.3	(n = 11)<br>3.7 ± 2.1	(n = 14)<br>**2.2 ± 1.2**	[1]NS<br>**[2]0.002**<br>**[3]0.011**
AFC	(n = 60)<br>22.8 ± 9.5	(n = 15)<br>23.0 ± 9.9	(n = 16)<br>20.5 ± 9.4	NS
Ovarian stimulation				
Total dose of FSH (IU)	2068 ± 720.9	2118 ± 681.7	*2488 ± 1197	[1]NS<br>[2]*0.062<br>[3]NS
Ovarian response				
Harvested oocytes	13.4 ± 6.4	**8.4 ± 5.2**	**9.7 ± 6.6**	**[1]0.001**<br>**[2]0.003**<br>[3]NS
Oocyte maturity rate (%)	79.9 ± 14.7	73.4 ± 23.2	**69.3 ± 31.9**	[1]NS<br>**[2]0.005**<br>[3]NS
Oocyte atretic rate (%)	8.13 ± 11.2	3.61 ± 5.83	4.97 ± 9.23	NS

Data are presented as the mean ± standard deviation. Bold values indicate statistically significant results, while asterisks (*) denote marginal differences ($0.05 \leq p \leq 0.1$). [1] Comparison OD vs. Grade 2; [2]OD vs. Grade 3; [3]Grade 2 vs. Grade 3. Statistical analyses were performed using ANOVA or Kruskal–Wallis test when assumptions for ANOVA were not met, and multivariate analyses adjusting for age and BMI were applied for AMH, AFC, ovarian stimulation and ovarian response parameters. AFC: Antral Follicle Count; AMH: Anti Mullerian Hormone; BMI: Body Mass Index; FSH: Follicle Stimulating Hormone; NS: Non significative; OD: Oocyte Donors.
