# Supplementary material for: Impact of Breast Cancer on Ovarian Function: Dysregulation of Cholesterol Homeostasis in Cumulus Cells and Follicular Fluid
Source: Cancers (Basel). 2026 May 1;18(9):1451. doi: 10.3390/cancers18091451 (PMC13163015; doi:10.3390/cancers18091451)
Supplement: Supplementary file 1 [file cancers-18-01451-s001.zip › Table S3.pdf]

Table S3: Characteristics of patients and ovarian response to stimulation according to lymph node invasion

	OD (n = 64)	Localised (n = 16)	Disseminated (n = 16)	p-value
Patient characteristics				
Age (years)	32.4 ± 3.9	**30.2 ± 3.0**	33.8 ± 2.9	[1]**0.045**<br>[2]NS<br>[3]**0.004**
BMI (kg/m[2])	23.4 ± 4.2	24.4 ± 5.1	23.0 ± 4.2	NS
AMH (ng/mL)	(n = 43)<br>3.9 ± 2.3	(n = 12)<br>3.3 ± 2.0	(n = 13)<br>*2.6 ± 1.4	[1]NS<br>[2*]0.091<br>[3]NS
AFC	(n = 60)<br>22.8 ± 9.5	(n = 15)<br>23.3 ± 9.8	(n = 15)<br>20.8 ± 9.4	NS
Ovarian stimulation				
Total dose of FSH (IU)	2068 ± 720.9	2322 ± 1146	2326 ± 893.7	NS
Ovarian response				
Harvested oocytes	13.4 ± 6.4	**9.25 ± 6.1**	**9.31 ± 6.0**	[1]**0.002**<br>[2]**0.004**<br>[3]NS
Oocyte maturity rate (%)	79.9 ± 14.7	*72.8 ± 30.1	**69.7 ± 26.9**	[1*]0.085<br>[2]**0.023**<br>[3]NS
Oocyte atretic rate (%)	8.13 ± 11.2	**4.51 ± 9.08**	4.47 ± 6.74	[1]**0.046**<br>[2]NS<br>[3]NS

Data are presented as the mean ± standard deviation. Bold values indicate statistically significant results, while asterisks (*) denote statistical trends. [1] Comparison OD vs. Localised cancer; [2]OD vs. Disseminated cancer; [3]Localised vs. Disseminated. Statistical analyses were performed using ANOVA or Kruskal–Wallis test when assumptions for ANOVA were not met, and multivariate analyses adjusting for age and BMI were applied for AMH, AFC, ovarian stimulation and ovarian response parameters. AFC: Antral Follicle Count; AMH: Anti Mullerian Hormone BMI: Body Mass Index; FSH: Follicle Stimulating Hormone; NS: Non significative; OD: Oocyte Donor.
