# Supplementary material for: Impact of Breast Cancer on Ovarian Function: Dysregulation of Cholesterol Homeostasis in Cumulus Cells and Follicular Fluid
Source: Cancers (Basel). 2026 May 1;18(9):1451. doi: 10.3390/cancers18091451 (PMC13163015; doi:10.3390/cancers18091451)
Supplement: Supplementary file 1 [file cancers-18-01451-s001.zip › Table S4.pdf]

Table S4: Characteristics of patients and ovarian response to stimulation according to BRCA gene mutated status

	OD (n = 64)	BRCA1 mutated (n = 7)	BRCA1 non mutated (n = 26)	p-value
Patient characteristics				
Age (years)	32.4 ± 3.9	**30.1 ± 1.6**	32.6 ± 3.6	**[1]0.023**<br>[2]NS<br>**[3]0.038**
BMI (kg/m^2)	23.4 ± 4.2	22.4 ± 2.7	23.6 ± 4.9	NS
AMH (ng/mL)	(n = 43)<br>3.9 ± 2.3	(n = 6)<br>2.9 ± 1.1	(n = 20)<br>*2.8 ± 1.9	[1]NS<br>[2]*0.066<br>[3]NS
AFC	(n = 60)<br>22.8 ± 9.5	(n = 6)<br>25.7 ± 11	(n = 25)<br>20.9 ± 9.1	NS
Ovarian stimulation				
Total dose of FSH (IU)	2068 ± 720.9	2196 ± 824.7	2382 ± 1057	NS
Ovarian response				
Harvested oocytes	13.4 ± 6.4	**11.0 ± 8.5**	**7.90 ± 3.6**	**[1]0.05**<br>**[2]<0.0001**<br>[3]NS
Oocyte maturity rate (%)	79.9 ± 14.7	**67.0 ± 30.9**	**70.8 ± 28.3**	**[1]0.03**<br>**[2]0.02**<br>[3]NS
Oocyte atretic rate (%)	8.13 ± 11.2	**0.68 ± 1.80**	*5.63 ± 8.55	**[1]0.017**<br>[2]NS<br>[3]*0.100

Data are presented as the mean ± standard deviation. Bold values indicate statistically significant results, while asterisks (*) denote statistical trends. BRCA1 mutated and non-mutated breast cancer are compared to the oocyte donor group and between them such as: [1] Comparison OD vs. BRCA1 mutated; [2]OD vs. BRCA1 non-mutated; [3] BRCA1 mutated vs. BRCA1 non-mutated. Statistical analyses were performed using ANOVA or Kruskal–Wallis test when assumptions for ANOVA were not met, and multivariate analyses adjusting for age and BMI were applied for AMH, AFC, ovarian stimulation and ovarian response parameters. AFC: Antral Follicle Count; AMH: Anti Mullerian Hormone BMI: Body Mass Index; FSH: Follicle Stimulating Hormone; NS: Non significative; OD: Oocyte Donors.
