# Supplementary material for: Impact of Breast Cancer on Ovarian Function: Dysregulation of Cholesterol Homeostasis in Cumulus Cells and Follicular Fluid
Source: Cancers (Basel). 2026 May 1;18(9):1451. doi: 10.3390/cancers18091451 (PMC13163015; doi:10.3390/cancers18091451)
Supplement: Supplementary file 1 [file cancers-18-01451-s001.zip › Table S5.pdf]

Table S5: p-values and effect sizes for the comparative analysis of the relative expression levels of cholesterol biosynthesis enzymes and regulators, and cholesterol biosynthesis intermediates concentrations, between oocyte donors and breast cancer molecular subgroups.

		OD vs. TN n = 12		OD vs. HR+ n = 6		OD vs. HER2+ n = 10	
		P value	Effect-size [95% CI]	P value	Effect-size [95% CI]	P value	Effect-size [95% CI]
**Enzymes**	**sqle_tbp**	0.63	-0.08 [-0.43; 0.26]	0.83	-0.04 [-0.40; 0.32]	**0.003**	**-0.54 [-0.89; -0.19]**
	**hmgcor_tbp**	0.33	0.17 [-0.18; 0.51]	0.46	-0.14 [-0.50; 0.22]	0.42	-0.14 [-0.49; 0.21]
	**cyp51_tbp**	0.54	0.11 [-0.24; 0.45]	0.15	0.26 [-0.10; 0.62]	*0.077	-0.31 [-0.66; 0.04]
	**lss_tbp**	0.66	-0.08 [-0.42; 0.27]	0.17	0.25 [-0.11; 0.61]	*0.085	-0.31 [-0.66; 0.04]
	**dhcr7_tbp**	*0.07 (n=11)	-0.32 [-0.67; 0.02]	0.83	0.04 [-0.32; 0.40]	**0.001**	**-0.60 [-0.95; -0.25]**
	**dhcr24_tbp**	0.26	0.20 [-0.15; 0.54]	0.24	0.22 [-0.14; 0.58]	0.24	-0.21 [-0.56; 0.14]
**Regulators**	**srebp2_tbp**	**0.027**	**0.39 [0.05; 0.73]**	**0.048**	**0.36 [0.01; 0.72]**	0.34	-0.17 [-0.52; 0.18]
	**insig_tbp**	0.23	0.21 [-0.14; 0.55]	0.27	0.20 [-0.16; 0.56]	0.40	-0.15 [-0.50; 0.20]
	**scap_tbp**	**0.003**	**0.54 [0.19; 0.88]**	**0.014**	**0.46 [0.10; 0.82]**	0.93	0.02 [-0.33; 0.37]
	**lxra_tbp**	**0.042**	**0.35 [0.01; 0.70]**	0.20	0.24 [-0.12; 0.60]	0.59	0.10 [-0.25; 0.44]
	**lxrb_tbp**	**0.035**	**0.37 [0.03; 0.71]**	*0.057	0.35 [-0.01, 0.71]	0.64	-0.08 [-0.43; 0.27]
**Intermediates**	**lanosterol**	0.16	0.25 [-0.10; 0.59]	**0.046**	**-0.37 [-0.73; -0.01]**	0.54	-0.11 [-0.46; 0.24]
	**ff_mas**	0.17	0.24 [-.10; 0.58]	0.48	-0.13 [-0.49; 0.23]	0.34	-0.17 [-0.52; 0.18]
	**desmosterol**	0.13	0.27 [-0.08; 0.61]	0.12	0.28 [-0.08; 0.64]	0.33	0.17 [-0.18, 0.52]
	**dihydrolanosterol**	0.34	0.16 [-0.18; 0.51]	0.47	-0.13 [-0.49; 0.23]	0.13	0.27 [-0.08; 0.62]
	**dihydro_ff_mas**	0.33	0.17 [-0.18; 0.51]	0.12	-0.29 [-0.65; 0.07]	*0.06	-0.31 [-0.68; 0.01]
	**dihydro_t_mas**	0.15	-0.25 [-0.60 ; 0.09]	**0.002**	**-0.59 [-0.95 ; 0.23]**	**0.008**	**-0.48 [-0.83 ; -0.13]**
	**lathosterol**	0.81	0.04 [-0.30 ; 0.39]	**<0.0001**	**-0.73 [-1.09 ; -0.37]**	**0.008**	**-0.48 [-0.83 ; -0.13]**
	**cholesterol**	0.48	-0.12 [-0.47 ; 0.22]	**<0.0001**	**-0.68 [-1.04 ; -0.32]**	0.27	-0.20 [-0.55 ; 0.15]

Bold p-values indicate statistically significant results ($p < 0.05$), while asterisks (*) denote marginal differences ($0.05 \leq p \leq 0.1$). OD: oocyte donors; TN: Triple Negative; HR+: Hormone-Receptor positive; HER2+: Human Epidermal growth factor Receptor-2 positive; HMGCoR: 3-hydroxy-3-methylglutaryl-coenzyme A reductase; SQLE: squalene epoxidase; LSS: lanosterol synthase; CYP51: lanosterol 14-α-demethylase; DHCR7: 7-dehydrocholesterol reductase;

DHCR24: 24-dehydrocholesterol reductase; SREBP2: Sterol Regulatory Element-Binding Protein-2; SCAP: Sterol regulatory element-binding protein cleavage-activating protein; INSIG: Insulin-induced genes; CI: confidence interval.
